# Aminoacyl-tRNA-dependent enzymes in natural product biosynthesis: structure-function insights

**DOI:** 10.1038/s41429-025-00893-w

**Published:** 2026-01-22

**Authors:** Yu Zheng, Yanhui Zhao, Shunji Takahashi

**Affiliations:** 1 RIKEN Center for Sustainable Resource Science, Saitama, Japan; 2 School of Environmental Science & Engineering, Shandong University, Qingdao, China

**Keywords:** Enzymes, Natural products

## Abstract

Aminoacyl-tRNAs, charged by aminoacyl-tRNA synthetases with cognate amino acids, are essential for protein synthesis in primary metabolism. Beyond this canonical role, increasing evidence highlights their involvement in natural product biosynthesis. In this review, we first describe the biosynthesis of the aminoacyl nucleoside sulfamate ascamycin from *Streptomyces* sp. 80H647, highlighting the discovery of the alanyl-tRNA synthetase-like enzyme AcmF through an AI-driven “Forecasting Biosynthesis” approach. Leveraging recent advances in AlphaFold 3, we constructed complex models of a broadened repertoire of aminoacyl-tRNA-dependent enzymes to provide preliminary structure-function insights. These include the isoleucyl-tRNA synthetase-like enzyme SbzA, Gcn5-related *N*-acetyltransferase-fold transferases, cyclodipeptide synthase family enzymes, and lantibiotic dehydratase-like peptide aminoacyl-tRNA ligases. The catalytic mechanisms of these aminoacyl-tRNA-dependent enzymes are summarized in detail in this review.

## Introduction

**Fig. 1:**
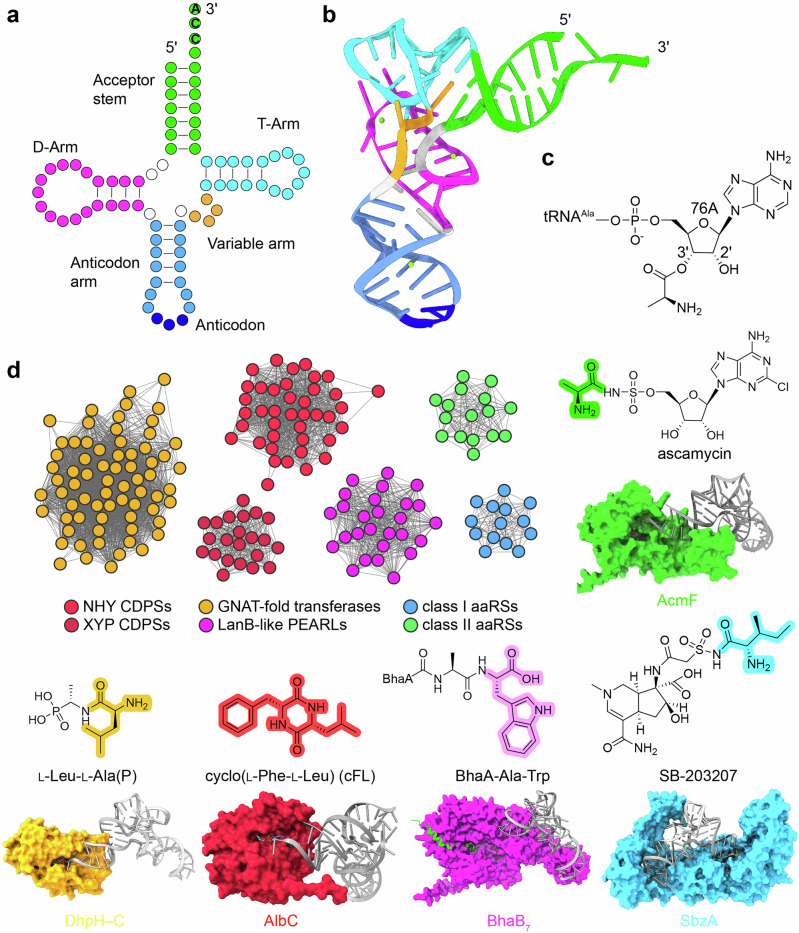
Structures of aminoacyl–tRNAs (aa–tRNAs) and their associated enzymes in natural product biosynthesis. **a** Representative cloverleaf secondary structure of the tRNA, with key structural elements highlighted in different colors. The essential 3’-end CCA sequence required for aminoacylation and protein synthesis is also indicated. **b** Three-dimensional structure of tRNA^Ala^ (PDB code 3WQY), shown as a cartoon representation. **c** Attachment of the alanyl moiety to the 3’-OH of A76 base at the 3’-end of tRNA^Ala^. **d** Sequence similarity network analysis of aa-tRNA-dependent enzymes involved in natural product biosynthesis. Examples of AlphaFold3–generated models (AcmF, DhpH–C, AlbC, BhaB_7_, and SbzA) in complex with small molecule and aa-tRNA substrates are shown

Aminoacyl-tRNAs (aa-tRNAs) are typically 70–100 nucleotides in length, with cognate amino acids covalently attached to the conserved 3’-end CCA sequence of the tRNA molecules (Fig. 1a–c) [1]. The amino acid is charged to the ribose of the terminal adenosine (A76) by aminoacyl–tRNA synthetases (aaRSs) through an ester bond, either at the 2’–hydroxyl group (by class I aaRSs, often followed by spontaneous transesterification to the 3’–OH) or directly at the 3’–OH group (by class II aaRSs) [2]. While aa–tRNAs are fundamental to primary metabolism, particularly in cellular processes such as protein synthesis, increasing evidence indicates that they also serve as aminoacyl donors in the biosynthesis of natural products (NPs) [3–6]. Notably, the diversity of biosynthetic enzymes that utilize aa–tRNAs as substrates has expanded [3–6], now encompassing members of Gcn5–related *N*-acetyltransferase (GNAT)-fold transferases, cyclodipeptide synthase (CDPS) family enzymes, class I lantibiotic dehydratase (LanB)-like enzymes, as well as class I and class II aaRS-like enzymes (Fig. 1d).

Despite recent advances in the discovery of novel biosynthetic enzymes, co-crystallization of enzymes in complex with aa–tRNAs and small molecule substrates remains challenging and time-consuming due to conformational changes upon binding and other reasons [7], limiting mechanistic insight into their catalytic activity. On the other hand, recent developments in artificial intelligence (AI) tools, like AlphaFold 3 [8] and RoseTTAFold All-Atom [9] have enabled the high–accuracy predictions of biomolecular complexes, including protein–protein, protein–small molecule, and protein–nucleic acid interactions. These advances offer powerful new avenues for the discovery and mechanistic analysis of aa-tRNA-dependent biosynthetic enzymes.

**Fig. 2:**
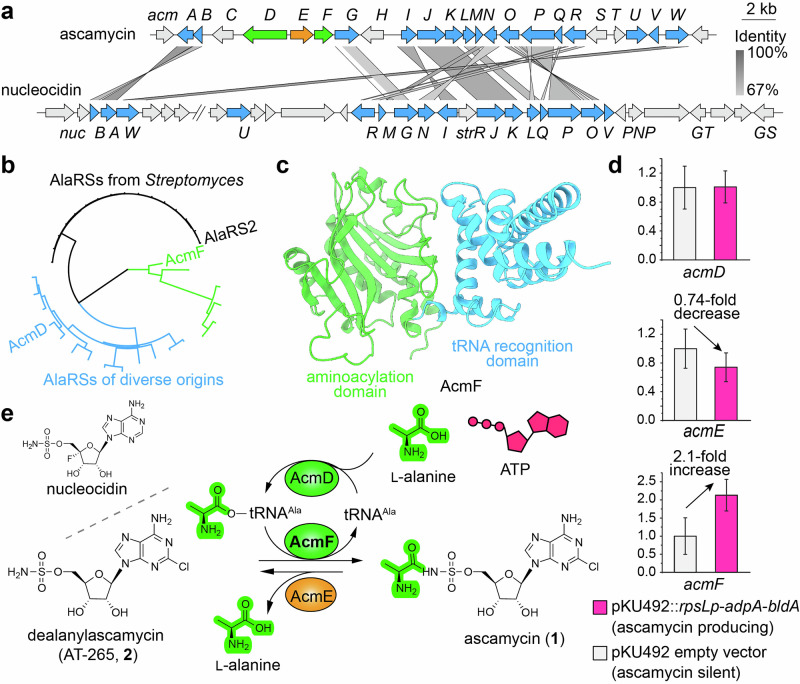
“Forecasting Biosynthesis”–guided discovery of AcmF as an aa–tRNA–dependent transferase in ascamycin biosynthesis. **a** Comparative analysis of the *acm* cluster from *Streptomyces* sp. 80H647 and *nuc* cluster from *Streptomyces calvus* ATCC 13382. Conserved genes are highlighted in blue. **b** Phylogenetic analysis of AcmD, AcmF, and the AlaRS2 annotated from *Streptomyces* sp. 80H647 genome. **c** Domain architecture of AcmF structure modeled by AlphaFold3. **d** Expression analysis of *acmD*, *acmE*, and *acmF* genes. *Streptomyces* sp. 80H647 strain was transformed with pKU492::*rpsLp*–*adpA*–*bldA* or the empty pKU492 vector as a control. Error bars represent standard deviation (s.d.) from three independent replicates. **e** Proposed reaction scheme showing AcmF–catalyzed aminoacylation converting dealanylascamycin (AT–265, **2**) to ascamycin (**1**). The structure of nucleocidin is also shown for comparison

In this review, we first present an AI-driven “Forecasting Biosynthesis” approach to identify a novel aa-tRNA-dependent enzyme in the biosynthesis of ascamycin (**1**) (Fig. 2). We also systematically discuss the preliminary structure–function relationships of the expanded repertoire of aa-tRNAs-dependent enzymes based on AlphaFold 3-modeled complex structures. All calculation experiments were conducted on a system equipped with an Intel i9-14900KF CPU (3.20 GHz, 4 physical cores), an NVIDIA RTX 6000 Ada GPU (48 GB VRAM), and 128 GB of installed memory.

## Discovery of alanyl-tRNA synthetase-like enzyme AcmF in ascamycin biosynthesis

Ascamycin (**1**) and dealanylascamycin (AT–265, **2**) are rare nucleoside sulfamates isolated from *Streptomyces* sp. 80H647 at RIKEN in the 1980s by Takahashi, Beppu, Isono, and co–workers (Fig. 2) [10, 11]. Subsequently, Osada and Isono found that both **1** and **2** inhibit protein synthesis in *Xanthomonas citri* and *Escherichia coli* by using cell-free systems, yet **1** only shows selective toxicity against *X. citri*. This is because **1** contains an l-alanine moiety bound to the 5’-*O*-sulfamate group of **2**, which restricts its permeation across bacterial membranes [12]. Later, Zhao et al. identified the biosynthetic gene cluster (*acm* cluster) responsible for producing **1** and **2** through gene disruption [13], although the assignment of genes responsible for *N*-alanylation and halogenation is questionable [14].

In our previous study [6], through comparative analysis with the *nuc* cluster form *Streptomyces calvus* ATCC 13382, which is responsible for nucleocidin biosynthesis (Fig. 2a), we selected three unique genes *acmD*, *acmE*, and *acmF* within the *acm* cluster that are putatively involved in the aa-tRNA-dependent conversion of **2** into **1**. A domain-based BLAST analysis revealed that AcmD and AcmF are alanyl-tRNA synthetase (AlaRS)-like proteins, likely responsible for attaching the alanyl moiety to **2** to produce **1**. In contrast, AcmE, an α/β–hydrolase fold superfamily protein, was hypothesized to catalyze the reverse reaction by cleaving the alanyl moiety from **1**. To explore the evolutionary relationship of these enzymes, we performed phylogenetic analysis on AcmD, AcmF, and AlaRS2, a canonical AlaRS enzyme annotated from the *Streptomyces* sp. 80H647 genome. AcmD clustered with AlaRS homologs from diverse bacterial lineages, whereas AcmF formed a distinct, independent clade (Fig. 2b), suggesting it represented a noncanonical AlaRS-like enzyme. Structural predictions of AcmD, AcmF, and AlaRS2 had been generated using ColabFold [15] and compared with deposited AlaRS crystal structures in the Protein Data Bank (PDB). Notably, AcmF was a truncated AlaRS-like protein containing only the *N*-terminal aminoacylation and tRNA recognition domains [16] (Fig. 2c), and it lacked most of the key residues required for adenosine triphosphate (ATP) binding and alanine activation [17]. Furthermore, two critical active-site mutations, N219Q and D232E, were identified in AcmF, disrupting the canonical aminoacyl–adenosine monophosphate (AMP) formation step [18], strongly indicating that AcmF lacks typical tRNA^Ala^ aminoacylation activity. In a parallel study, we had introduced and constitutively expressed two global transcriptional regulators *adpA* and *bldA*, in *Streptomyces* sp. 80H647 to enhance the in vivo conversion of **2** into **1**. Gene expression analysis showed a 2.1-fold upregulation of *acmF* compared to the vector control (Fig. 2d). Collectively, these combined bioinformatics, AI-based, and experimental data strongly supported AcmF as the candidate enzyme responsible for catalyzing aminoacylation of **2** to form **1** via an aa–tRNA–dependent manner.

We had successfully purified recombinant AcmD, AcmE, and AcmF and verified their enzymatic activities through in vitro assays. AcmE, which contains an *N*-terminal signal peptide sequence, is predicted to be a secretory hydrolase, cleaving **1** into **2** and l-alanine (Fig. 2e). Most importantly, AcmF catalyzed the aminoacylation of **2** to produce **1**, utilizing Ala–tRNA^Ala^ produced by AcmD through canonical tRNA^Ala^ aminoacylation reaction using l-alanine and ATP [6]. In addition, we had uncovered the self–resistance mechanism of the producer strain *Streptomyces* sp. 80H647, in which the additional copy of AlaRS, AcmD in the *acm* cluster, exhibited greater resistance to the AcmF-produced **1** than the canonical AlaRS enzyme [6].

## Mechanistic study of AcmF based on AlphaFold 3-predicted models

**Fig. 3:**
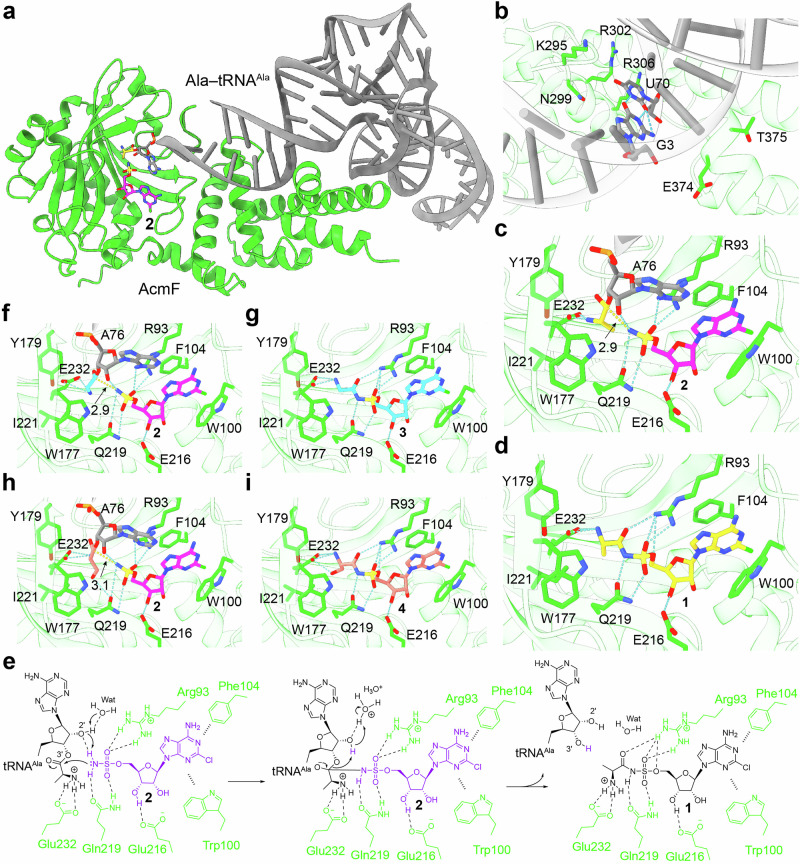
Structure–function insights into the AlaRS–like enzyme AcmF–catalyzed reaction. **a** The AlphaFold3–generated complex model of AcmF bound to dealanylascamycin (AT–265, **2**) and Ala–tRNA^Ala^ (GGC) substrates. The 3’–end A76 of tRNA^Ala^ (GGC) charged with an alanyl moiety at the 3’–OH is shown as sticks. **b** Close-up view of AcmF tRNA recognition domain. The G3:U70 base pair of Ala–tRNA^Ala^ (GGC) is depicted as sticks. **c**,**d** Close-up views of the AcmF active site. AcmF residues surrounding the substrates **2** and Ala–tRNA^Ala^ (GGC) (**c**), or the product ascamycin (**1**) (**d**), are depicted as sticks. Hydrogen bonds and distances (Å) are represented by cyan and yellow dashed lines, respectively. **e** The proposed catalytic mechanism of AcmF based on the AlphaFold3–generated complex model in this review and the proton relay mechanism in aaRSs [20, 21]. Wat, water molecule. Structure basis of AcmF activity in producing glycyl–ascamycin (**3**). AcmF residues surrounding the substrates **2** and Gly–tRNA^Ala^ (GGC) (**f**), or the product **3** (**g**), are depicted as sticks. Structure basis of AcmF activity in producing seryl–ascamycin (**4**). AcmF residues surrounding the substrates **2** and Ser–tRNA^Ala^ (GGC) (**h**), or the product **4** (**i**), are depicted as sticks. Hydrogen bonds and distances (Å) are represented by cyan and yellow dashed lines, respectively

Next, we had sought to investigate the catalytic mechanism of AcmF in our previous study by performing molecular docking of the substrate **2** into the ColabFold–predicted structure of the enzyme [6], although we were unable to capture the interaction between AcmF and its second substrate, Ala–tRNA^Ala^. To further probe the catalytic mechanism, we had performed alanine substitutions of candidate residues within the catalytic cavity and identified Glu232 as a key residue essential for enzymatic activity [6]. In this review, we successfully generated the complex model of AcmF, **2**, and Ala-tRNA^Ala^ using AlphaFold 3, enabling a comprehensive and detailed mechanistic study of the enzyme. AcmF is predicted to form a complex with the Ala–tRNA^Ala^ substrate (Fig. 3a), resembling the interaction observed in the crystal structure of the *Archaeoglobus fulgidus* AlaRS bound to tRNA^Ala^ and an alanyl-adenylate analog (Ala-AMS) (PDB code 3WQY) [16]. However, AcmF does not appear to selectively recognize aa-RNA substrates, likely due to the absence of the Asp-plus-Asn architecture that typically recognizes the G3:U70 pair of tRNA^Ala^ [19] (Fig. 3b), although an E374A mutation in tRNA recognition domain resulted in a 76% reduction in enzymatic activity [6]. In a close–up view (Fig. 3c), **2** is positioned within the aminoacylation active site of AcmF, occupying a location similar to that of Ala–AMSs observed in the crystal structures of *A. fulgidus* AlaRS (PDB code 3WQY) and *E. coli* AlaRS (PDB code 3HXU) [16, 18]. Specifically, AcmF stabilizes **2** through π–π stacking interactions with Trp100 and Phe104, and network of hydrogen bonds with Arg93, Glu216, and Gln219. In addition, AcmF holds the alanyl moiety of the Ala–tRNA^Ala^ substrate through Glu232, facilitating the aminoacyl transfer onto **2** to produce **1** (Fig. 3c,d). Notably, hydrogen bond formation is also observed among the sulfamate group of **2** and the 2’–OH group of Ala–tRNA^Ala^ in the AlphaFold 3-predicted model (Fig. 3c).

Based on the predicted interactions and the previously reported proton relay mechanism in aaRSs [20, 21], we speculate a refined catalytic mechanism for AcmF in this review (Fig. 3e). First, **2** binds in the active site, where the oxygen atoms of its sulfamate group (–O–SO_2_–NH_2_) form hydrogen bonds with Arg93 and Gln219, anchoring the molecule and helping to polarize the –NH_2_ group. This network, together with the 2’–OH group of Ala–tRNA^Ala^, positions the nucleophile for activation. Proton abstraction from the –NH_2_ group is likely facilitated through a 2’–OH-mediated proton relay that may involve additional structured water molecules. Then, the activated –NH_2_ group attacks the ester carbonyl carbon of the alanyl moiety linked to the tRNA 3’–O atom, forming a tetrahedral intermediate. A hydrogen bond between Glu232 and the alanyl–NH_2_ group may help orient and stabilize the transition state. Next, collapse of the tetrahedral intermediate forms the new amide bond in **1** and releases the tRNA. Finally, the proton originally abstracted from the –NH_2_ of **2** is most likely transferred to the tRNA 3’–O atom, either directly from the 2’–OH or indirectly via a water–mediated pathway, regenerating the free 3’–OH group of tRNA. This mechanism highlights how AcmF adapts AlaRS–like structural motifs to catalyze an unusual aminoacyl transfer reaction in **1** biosynthesis.

In addition, we had previously reported that AcmF can accept the Gly-tRNA^Ala^ and Ser–tRNA^Ala^ substrates, which are mischarged by AcmD, to produce glycyl-ascamycin (**3**) and seryl-ascamycin (**4**) derivatives [6]. In this review, we also generated the complex models of AcmF bound to **2** and Gly-tRNA^Ala^ (Fig. 3f), product **3** (Fig. 3g), **2** and Ser-tRNA^Ala^ (Fig. 3h), and product **4** (Fig. 3i), and observed interactions similar to those described above. Taken together, these experimental data and AI–based analyses suggest that AcmF may represent a promising chemoenzymatic platform for the synthesis of various aminoacyl nucleoside sulfamates.

## The isoleucyl-tRNA synthetase-like enzyme SbzA in SB-203208 biosynthesis

**Fig. 4:**
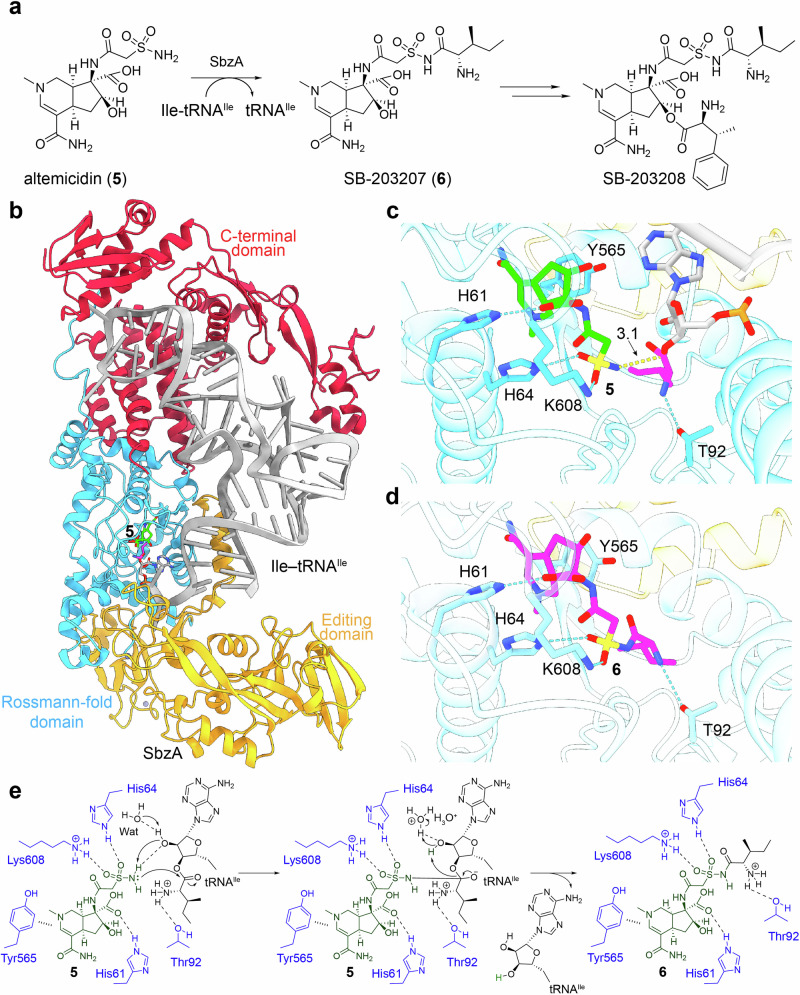
Structure–function insights into IleRS-like enzyme SbzA in SB–203208 biosynthesis. **a** The SbzA-catalyzed aminoacyl transfer reaction in sulfonamide SB–203207 (**6**) biosynthesis. **b** The AlphaFold3–generated complex model of IleRS-like SbzA bound to sulfonamide altemicidin (**5**) and Ile–tRNA^Ile^ (GAT). The 3’-end A76 of tRNA^Ile^ (GAT) charged with an isoleucyl moiety at the 3’–OH is shown as sticks. Close-up views of the SbzA active site. SbzA residues surrounding **5** and Ile–tRNA^Ile^ (GAT) (**c**), or the product **6** (**d**), are depicted as sticks. Hydrogen bonds and distances (Å) are represented by cyan and yellow dashed lines, respectively. **e** The proposed catalytic mechanism of SbzA based on the AlphaFold3–generated complex model in this review and proton relay mechanism in aaRSs [20, 21]. Wat water molecule

Altemicidin (**5**), SB–203207 (**6**), and SB–203208 are rare naturally occurring sulfonamides isolated from *Streptomyces* spp. (Fig. 4a), identified respectively as an acaricidal and antitumor agent, and as an isoleucyl–tRNA synthetase (IleRS) inhibitor [5, 22–25]. Later, Yan et al. applied a resistance gene–guided genome mining approach and identified the *sbz* cluster responsible for the production of **5** and **6** in *Streptomyces* sp. NCIMB40513 [26]. Interestingly, Hu et al. found that the self–resistance protein SbzA, an additional IleRS copy encoded within the *sbz* cluster, utilizes Ile–tRNA^Ile^ produced by canonical Ssp–IleRS enzyme to install the isoleucyl moiety onto **5** to produce **6** [5]. They had also reported that SbzA exhibits weak yet detectable canonical tRNA aminoacylation activity, producing trace amounts of Ile–tRNA^Ile^ that can be used for the aminoacylation of **3** [5].

**Fig. 5:**
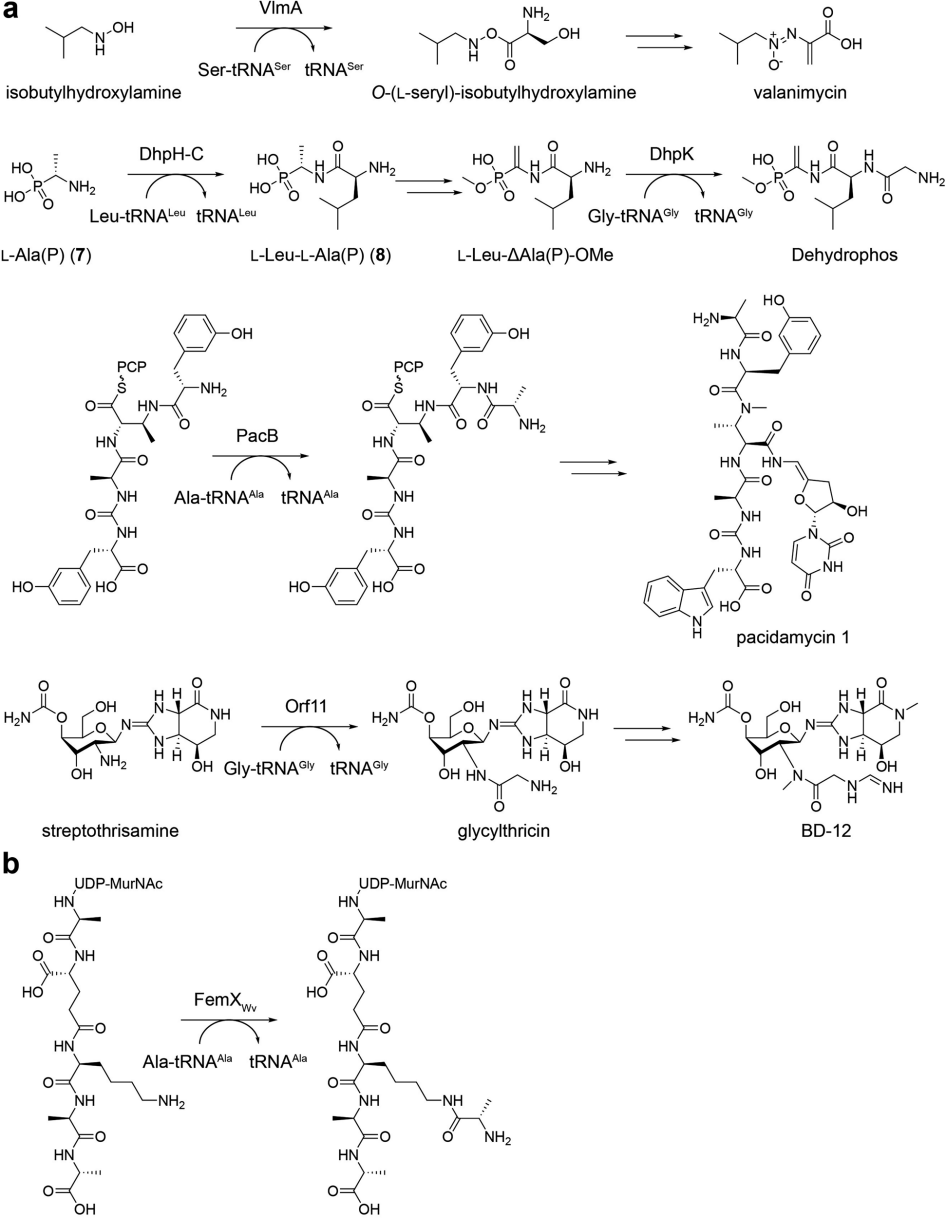
GNAT-fold transferases-catalyzed reactions in natural product biosynthesis. **a** Representative roles of GNAT-fold transferases in natural product biosynthesis, including VlmA, DhpH–C, DhpK, PacB, and Orf11. **b** FemX_Wv_ from *Weissella viridescens*, involved in bacterial cell wall peptidoglycan synthesis

In this review, we successfully constructed an AlphaFold 3-based complex model of SbzA bound with **5** and Ile-tRNA^Ile^ to gain preliminary structure–function insights (Fig. 4b). Overall, SbzA retains the canonical IleRS protein architecture, including a Rossmann-fold domain for aminoacylation, an editing domain for post-transfer editing, and a C-terminal domain contributing for tRNA^Ile^ binding [27]. In the close-up view (Figs. 4c), **5** occupies the aminoacylation active site of SbzA, in a position similar to mupirocin and Ile-AMS observed in the crystal structures of *Priestia megaterium* IleRS2 (8C8U and 8C8V) [28]. SbzA stabilizes **5** through π–π stacking interaction with Tyr565, and a hydrogen-bonding network involving His61, His64, and Lys608 on the signature motifs ^61^HYGH^64^ and ^605^AMSKA^609^ [28]. In addition, SbzA likely interacts with the isoleucyl moiety of Ile–tRNA^Ile^ through hydrogen–bonding with Thr92 (Fig. 4c), thereby facilitating its transfer onto **5** to generate **6** (Fig. 4d).

Based on the predicted complex structure and parallels with the proton relay mechanisms of aaRSs and AcmF, we propose a possible catalytic mechanism for SbzA in this review (Fig. 4e). First, **5** binds in the active site, where the sulfonamide group (–SO_2_–NH_2_) forms hydrogen bonds with His64 and Lys608, anchoring the substrate and polarizing the –NH_2_ group. Structured water molecules likely mediate local proton abstraction from the –NH_2_ group, activating it for nucleophilic attack. Then, the deprotonated –NH_2_ group attacks the ester carbonyl carbon of the isoleucyl moiety linked to the tRNA 3’–OH of tRNA. A stabilizing hydrogen bond between Thr92 and the isoleucine –NH_2_ group may help orient the reacting groups during this process. This leads to the formation of a tetrahedral intermediate, which collapses to form a new amide bond in **6** while releasing the tRNA. Finally, the proton originally abstracted from the –NH_2_ of **5** is most likely transferred to the departing 3’–O atom of tRNA through a water–mediated pathway, regenerating the free 3’–OH group.

## The GNAT-fold transferases in diverse natural product biosynthesis

The GNAT–fold superfamily encompasses a diverse group of aa-tRNA-dependent transferases involved in natural product biosynthesis, including VlmA in the valanimycin pathway [29], DhpH and DhpK in the dehydrophos pathway [30], PacB in pacidamycins biosynthesis [31], and Orf11 in the biosynthesis of the streptothricin analog BD–12 [32] (Fig. 5a). Valanimycin is an azoxy-containing antibiotic isolated from *Streptomyces viridifaciens* MG456-hF10 broth during a prescreening for antitumor compounds [33]. Garg et al. identified VlmA as the essential enzyme that converts isobutylhydroxylamine to *O*–(l–seryl)–isobutylhydroxylamine, based on feeding experiments with [^32^P]–labeled Ser–tRNA^Ser^ and in vitro enzymatic characterization [29]. Dehydrophos is a broad–spectrum phosphonate tripeptide antibiotic produced by *Streptomyces luridus* NRRL 15101 [34]. Bougioukou and co–workers reconstructed its biosynthesis in vitro and revealed a tRNA–dependent pathway, showing that the C–terminal domain of DhpH (DhpH–C) converts l–Ala(P) (**7**) to l–Leu–l–Ala(P) (**8**), while DhpK converts l–Leu–ΔAla(P)–OMe to dehydrophos using Leu–tRNA^Leu^ and Gly–tRNA^Gly^, respectively [30]. Pacidamycins are uridyl peptide antibiotics assembled by *Streptomyces coeruleorubidus* strains via a nonribosomal peptide synthetase pathway [35, 36]. Zhang et al. later elucidated that PacB introduces l-alanine to the growing peptidyl chain through genetic deletion and enzymatic analysis [31]. BD–12, a streptothricin-related antibiotic, was isolated from *Streptomyces luteocolor* NBRC 13826 [37]. Maruyama et al. identified Orf11 as a Gly-tRNA^Gly^-dependent enzyme that converts streptothrisamine into glycylthricin in the BD–12 pathway, validated through in vitro analysis [32]. Notably, VlmA, DhpH–C, DhpK, PacB, and Orf11 share amino acid sequence and structural similarities with FemABX family peptidyl transferases involved in bacterial cell wall peptidoglycan synthesis, such as FemX_Wv_ from *Weissella viridescens* (Fig. 5b) [38].

**Fig. 6:**
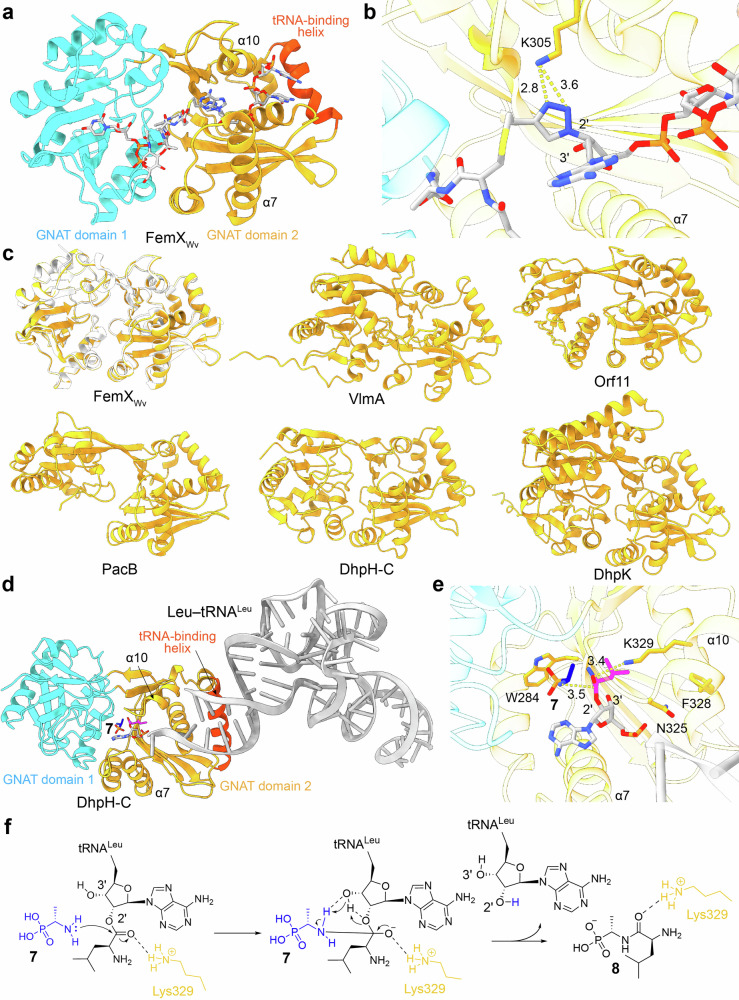
Structure–function insights into GNAT-fold transferases. **a** Crystal structure of FemX_Wv_ bound to a peptidyl-RNA conjugate (sticks) (PDB code 4II9). **b** Close–up views of the FemX_Wv_ active site highlighting the catalytic Lys305 residue (sticks). Distances (Å) are shown as yellow dashed lines. **c** AlphaFold 3–predicted structures of VlmA, DhpH–C, DhpK, PacB, Orf11. The predicted structure of FemX_Wv_ is aligned with the crystal structure (PDB code 4II9) for comparison. **d** AlphaFold 3-generated complex model of DhpH–C bound to l–Ala(P) (**5**) and Leu–tRNA^Leu^ (TAG). The leucyl moiety charged at the tRNA 2’–OH is shown as sticks. **e** Close-up views of the DhpH–C active site showing catalytic Lys329 and key residues Trp284, Asn325, and Phe328 (sticks). Distances (Å) are shown as yellow dashed lines. **f** The proposed catalytic mechanism for DhpH-C based on AlphaFold3–generated complex model in this review and substrate–assisted proton shuttling mechanism for GNAT–fold transferases [40, 43]

The structures of FemX_Wv_ (PDB codes 1NE9, 4II9), and FemX_Sa_ (PDB code 6SNR) and FemA_Sa_ (PDB code 1LRZ) from *Staphylococcus aureus* were solved by X-ray crystallography in previous studies [38–41]. FemX_Wv_ adopted a double-GNAT-fold structure, with its two GNAT domains forming a binding site for the UDP-MurNAc-pentapeptide substrate (Fig. 6a) [38, 40]. In addition, FemX_Wv_ was proposed to interact with the acceptor stem of tRNA via a positively charged helix on the C-terminal GNAT domain 2 (Fig. 6a) [42]. Since Lys305 was the only reactive residue near the substrate and no classical catalytic base or acid is present (Fig. 6b), FemX_Wv_ was thought to catalyze aminoacyl transfer from the 2’–OH position of Ala-tRNA^Ala^ through a substrate-assisted proton shuttling mechanism [40, 43].

As no crystal structures are available, we modeled the protein structures here to gain preliminary structure–function insights. VlmA, DhpH–C, DhpK, PacB, and Orf11 showed common protein architecture with FemX_Wv_, each containing two GNAT domains, a substrate–binding site between them, and a tRNA–binding helix (Fig. 6c). Using DhpH–C as an example, we constructed a complex model bound with **7** and Leu–tRNA^Leu^ (Fig. 6d). DhpH–C forms a tRNA 3’–end access channel between helices α7 and α10, and likely interacts with the leucyl moiety of Leu–tRNA^Leu^ through hydrogen–bonding with a conserved Lys329 residue, analogous to Lys305 in FemX_Wv_ (Fig. 6e). Notably, Ulrich et al. verified the essential role of Lys329, as well as the contributions of Trp284, Asn325, and Phe328, for DhpH-C activity through alanine mutagenesis, supporting their roles in substrate interaction [44]. Taking together the structural similarity with FemX_Wv_, our predicted complex model, and biochemical validation [44], we propose in this review that the GNAT–fold transferase DhpH-C catalysis proceeds via a substrate-assisted proton shuttling mechanism like that of FemX_Wv_ (Fig. 6f).

## The CDPSs in cyclodipeptide natural product biosynthesis

**Fig. 7:**
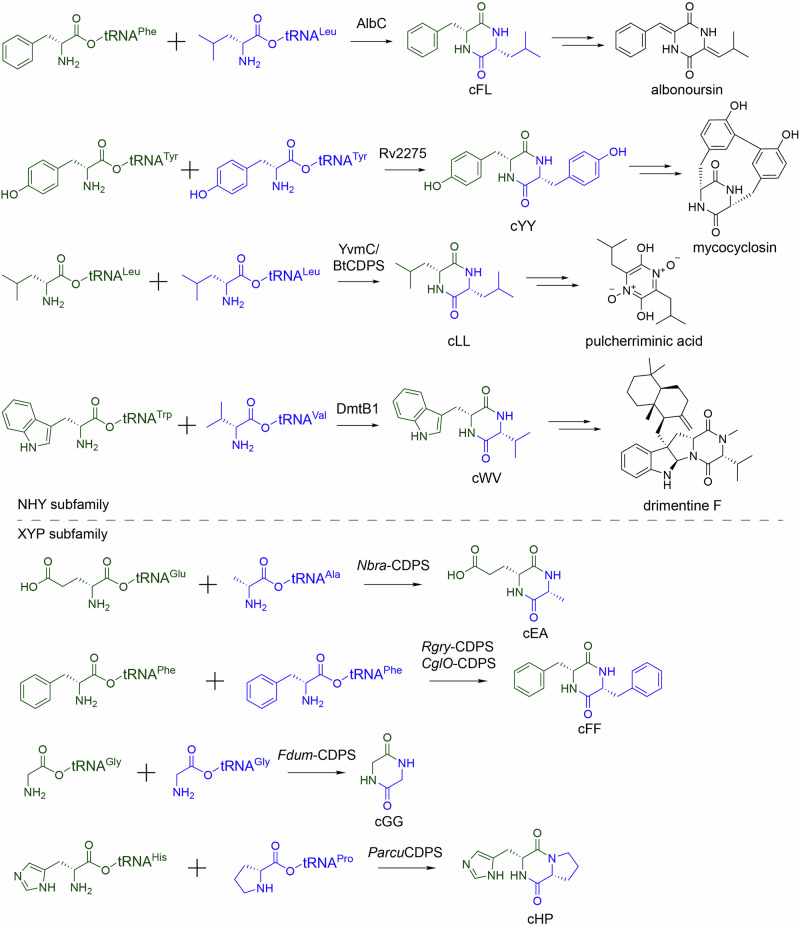
CDPSs-catalyzed cyclodipeptide formation in natural product biosynthesis

The CDPSs use two aa–tRNA substrates to generate diverse cyclodipeptide scaffolds for natural product biosynthesis (Fig. 7). Phylogenetically, the CDPSs are classified into two main subfamilies, NYH and XYP, based on the conserved N–Y–H and X–Y–P motifs that determine substrate specificity [45]. The NYH subfamily includes the first characterized CDPS AlbC from *Streptomyces noursei*, which uses Phe–tRNA^Phe^ and Leu–tRNA^Leu^ in the albonoursin pathway, although it can use a second Phe–tRNA^Phe^ substrate [46]; Rv2275 from *Mycobacterium tuberculosis*, which uses two Tyr–tRNA^Tyr^ substrates in the mycocyclosin pathway [47]; YvmC and BtCDPS from *Bacillus* species, which both use two Leu–tRNA^Leu^ substrates in the biosynthesis of pulcherriminic acid [47, 48]; and DmtB1 from *Streptomyces youssoufiensis* OUC6819, which uses Trp–tRNA^Trp^ and Val–tRNA^Val^ in the drimentine F pathway [49], among others. The XYP subfamily comprises *Nbra*–CDPS from *Nocardia brasiliensis* using Glu–tRNA^Glu^ and Ala–tRNA^Ala^ [50]; *Rgry*–CDPS from *Rickettsiella grylli* using Phe–tRNA^Phe^ and Phe–tRNA^Phe^ or Leu–tRNA^Leu^ [50]; *Fdum*–CDPS from *Fluoribacter dumoffii* using two Gly–tRNA^Gly^ substrates [50]; *CglO*–CDPS from *Candidatus* Glomeribacter gigasporarum using two Phe–tRNA^Phe^ substrates [51]; and *Parcu*CDPS from *Parcubacteria* bacterium RAAC4_OD1_1 using His–tRNA^His^ and Pro–tRNA^Pro^ [52], among others.

**Fig. 8:**
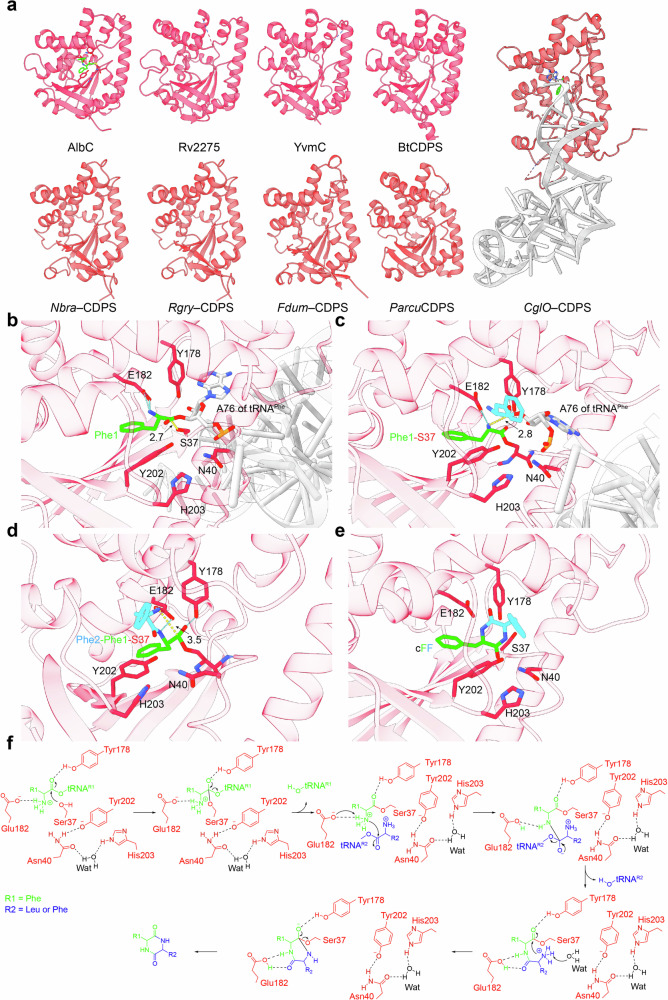
Structure–function insights into CDPSs. **a** Crystal structures of AlbC S37C–ZPK covalent intermediate (sticks) (PDB code 4Q24), Rv2275 (PDB code 2X9Q), YvmC (PDB code 3OQH), BtCDPS (PDB code 6ZU3), *Nbra*–CDPS (PDB code 5MLQ), *Rgry*–CDPS (PDB code 5MLP), *Fdum*–CDPS (PDB code 5OCD), *Parcu*CDPS (PDB code 7QB8), and *CglO*–CDPS in complex with Phe–tRNA^Phe^ (PDB code 6Y4B). Close–up views of the AlbC active site from AlphaFold 3-predicted complex models: **b** AlbC bound with the first Phe–tRNA^Phe^, **c** Phenylalanyl–AlbC bound with the second Phe–tRNA^Phe^, **d** Diphenylalanyl–AlbC intermediate, **e** AlbC bound with the cyclized diphenylalanyl product. AlbC active site residues are shown as sticks. Hydrogen bonds and distances (Å) are represented by cyan and yellow dashed lines, respectively. **f** Conserved ping–pong catalytic mechanism of CPDSs, with AlbC shown as a representative example based on AlphaFold3–generated complex model in this review and a previously reported crystal structure (PDB code 4Q24). In this model, Tyr202 does not appear to participate in the final nucleophilic attack, differing from the mechanism proposed in a previous publication [54]. Wat, water molecule

To date, extensive efforts employing protein crystallography and biochemical characterization have been made to elucidate the catalytic mechanisms of CDPSs. For example, Vetting et al. reported the first crystal structure of a CDPS, Rv2275, solved at 2.0 Å high resolution (Fig. 8a) (PDB code 2X9Q) [53]. Interestingly, they had found that the Rossmann–fold structure of Rv2275 is highly similar to the catalytic domains of tyrosyl– and tryptophanyl–tRNA synthetases (class I aaRSs), suggesting a potential evolutionary relationship [53]. Notably, they had confirmed the formation of a covalent aminoacyl–enzyme intermediate, in which the tyrosyl moiety is transferred from the Tyr–tRNA^Tyr^ substrate to the active site Ser88. Later, crystal structures of AlbC (PDB code 3OQV) and a S37C variant covalently bound to *N*–carbobenzyloxy–l–Phe–methyl ketone (ZPK) intermediate analog (PDB code 4Q24) had been solved. Moutiez et al. had further demonstrated the formation of the covalent dipeptidyl–AlbC intermediate, reinforcing the conserved ping–pong mechanism of CDPSs [54]. In addition, the crystal structures of YvmC (PDB codes 3OQH, 3OQI, 3OQJ, 3S7T), BtCDPS (PDB codes 6ZTU, 6ZU3, 7AZU), *Nbra*–CDPS (PDB code 5MLQ), *Rgry*–CDPS (PDB code 5MLP), *Fdum*–CDPS (PDB code 5OCD), *CglO*–CDPS in complex with Phe–tRNA^Phe^ (PDB code 6Y4B), and *Parcu*CDPS (PDB code 7QB8) had also been reported (Fig. 8a). Despite differences in subfamily classification, all CDPSs share the conserved Rossmann–fold architecture and have been thought to catalyze cyclodipeptide formation through a conserved mechanism [45, 54, 55].

In this review, we use the well-studied AlbC as an example to illustrate the reported catalytic mechanism [54], through AlphaFold 3-constructed complex models. In stage 1, the phenylalanyl moiety of the first Phe–tRNA^Phe^ substrate binds to AlbC, where hydrogen bonds with active-site residues such as Glu182 position it for nucleophilic attack by the hydroxyl group of the catalytic Ser37 (Fig. 8b). This attack on the ester carbonyl carbon of Phe–tRNA^Phe^ forms a phenylalanyl-AlbC intermediate. In stage 2, Glu182 likely functions as a general base, facilitating nucleophilic attack of the first phenylalanyl moiety on the ester carbonyl carbon of the second Phe–tRNA^Phe^ substrate (Fig. 8c), yielding a diphenylalanyl-AlbC intermediate. In stage 3, the dipeptidyl moiety is activated by structured water in active site (Fig. 8d), enabling nucleophilic attack on the enzyme ester bond. Finally, in stage 4, this reaction releases the cyclized diphenylalanyl product from AlbC (Fig. 8e). Collectively, these modeled complexes provide structural insights supporting the conserved ping-pong mechanism of CDPSs (Fig. 8f).

## The LanB-like peptide aminoacyl-tRNA ligases (PEARLs)

**Fig. 9:**
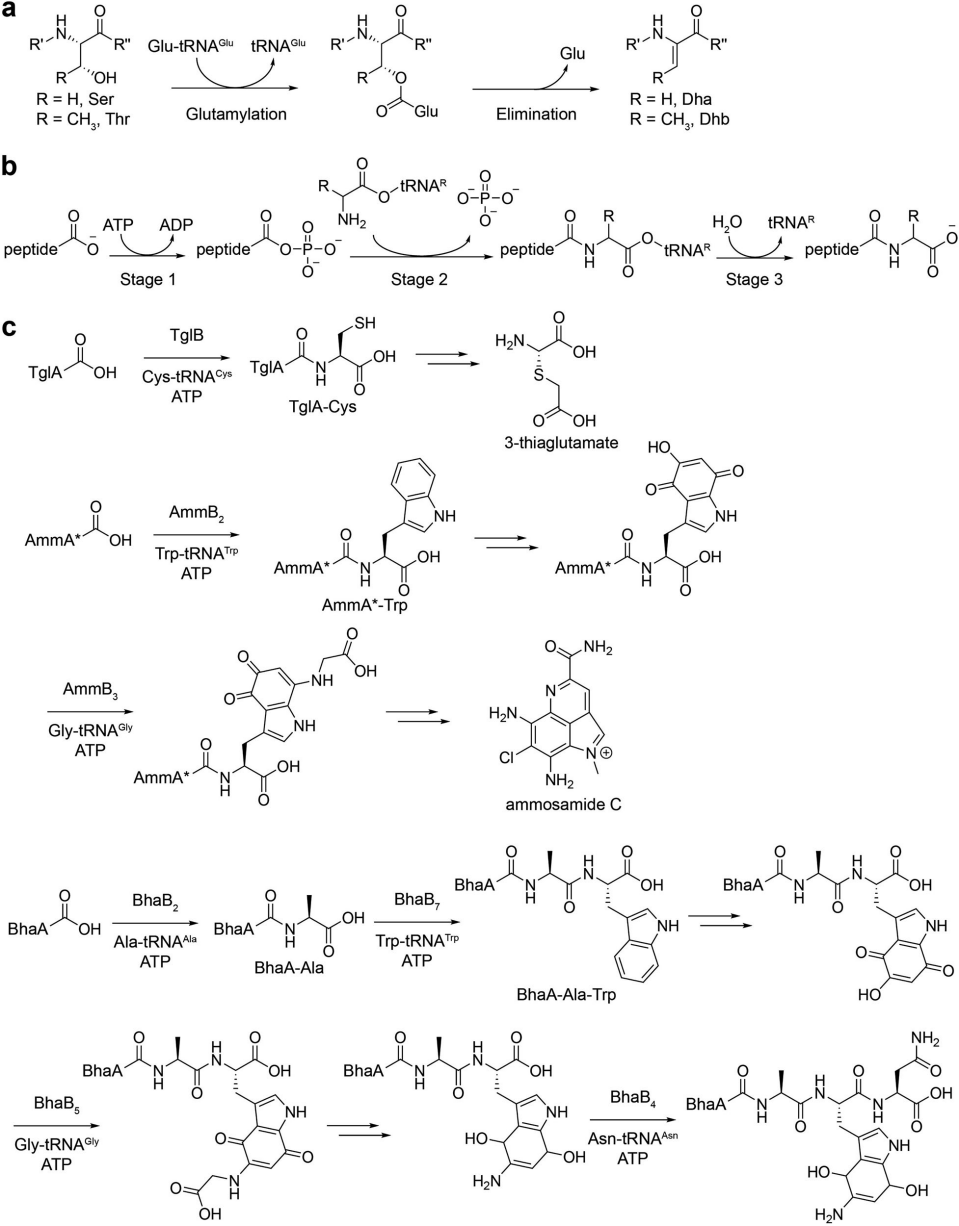
LanB-like enzymes-catalyzed aminoacyl transfer reactions. **a** Glu-tRNA^Glu^-dependent dehydration reactions catalyzed by LanB enzymes during class I lantibiotic biosynthesis. **b** ATP– and aa–tRNA–dependent C-terminal peptide extensions catalyzed by LanB–like PEARLs. **c** Representative examples of PEARLs–catalyzed aminoacyl transfer reactions

PEARLs are evolutionarily related to canonical LanBs but perform distinct functions [56]. Canonical LanBs, such as NisB from *Lactococcus lactis* in the nisin pathway and MibB from *Microbispora* sp. 107891 in the microbisporicin A1 pathway [57, 58], utilize Glu-tRNA^Glu^ as a co-substrate to catalyze sequential glutamylation and glutamate elimination reactions, thereby dehydrating serine and threonine residues on precursor peptides during class I lantibiotic biosynthesis (Fig. 9a). In contrast, PEARLs, reported mainly by van der Donk and co-workers, are small LanB-like proteins that catalyze C–terminal extensions of precursor peptides in an ATP- and aa–tRNA-dependent manner (Fig. 9b) [56, 59–61]. Examples include: TglB from *Pseudomonas syringae* pv. maculicola ES4326, which installs a cysteine to TglA using Cys–tRNA^Cys^ in 3-thiaglutamate biosynthesis [56, 59]; AmmB_2_ from *Streptomyces* sp. CNR698, which installs a tryptophan to AmmA*, followed by glycine attachment to the indole ring of the modified AmmA*–Trp by AmmB_3_ in ammosamide C biosynthesis [56, 60]; and the orthologues from *Bacillus halodurans* C–125, including BhaB_1_ (alanine installation on BhaA), BhaB_7_ (tryptophan installation on BhaA–Ala), BhaB_5_ (glycine attachment to the indole ring), and BhaB_4_ (asparagine installation on the modified BhaA–Ala–Trp) [60–62], among others (Fig. 9c). Despite their diverse origins, the PEARLs are proposed to share a common catalytic mechanism (Fig. 9b). In stage 1, the PEARLs activate the C–terminal carboxylate of precursor peptides through ATP–dependent phosphorylation. In stage 2, –NH_2_ group of the cognate aa–tRNA performs nucleophilic attack on the activated carbonyl carbon, forming a new amide bond. In stage 3, an active site water molecule hydrolyzes the peptide–tRNA intermediate, releasing the extended peptide and free tRNA.

**Fig. 10:**
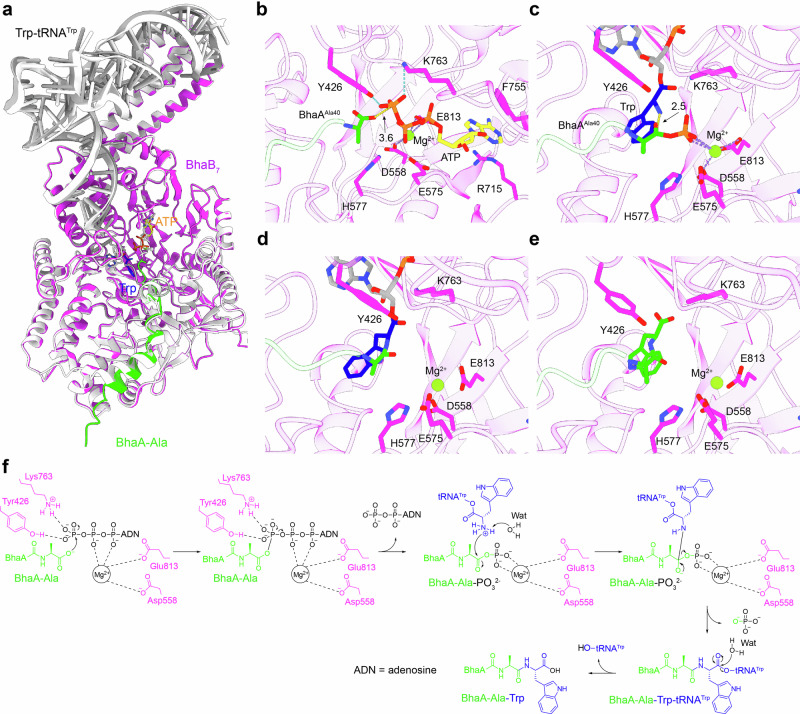
Structure–function insights into PEARLs using BhaB_7_ as an example. **a** Alignment of the BhaB_7_ complex structure modeled here (bound with BhaA–Ala, ATP, and Trp–tRNA^Trp^) with the structure reported by van der Donk and co-workers (white, bound with BhaA–Ala, ATP, and tRNA^Trp^). Close–up views of the BhaB_7_ active site from AlphaFold 3–predicted complex models bound with: **b** BhaA–Ala and ATP, **c** C–terminal phosphorylated BhaA–Ala and Trp–tRNA^Trp^, **d** BhaA–Ala–Trp–tRNA^Trp^ intermediate, **e** The extended BhaA–Ala–Trp product. BhaB_7_ active site residues are shown as sticks. Hydrogen bonds and distances (Å) are represented by cyan and yellow dashed lines, respectively. **f** The proposed possible catalytic mechanism of BhaB_7_ based on AlphaFold3–generated complex model in this review. Wat water molecule

Since no crystal structures of PEARLs have been reported, we used BhaB_7_ as a representative example to model its complex with ligands and gain preliminary structure–function insights. The BhaB_7_ structure modeled here in complex with BhaA–Ala, ATP, and Trp-tRNA^Trp^ closely resembles the recently reported AlphaFold 3-based model of BhaB_7_ in complex with BhaA–Ala, ATP, and tRNA^Trp^ by van der Donk et al. [63] (Fig. 10a). In stage 1, BhaB_7_ is predicted to bind ATP and Mg^2+^ ion through interactions with Arg34, Tyr426, Glu575, Asp558, His577, Arg715, Phe755, Lys763, Glu813, Arg815, thereby activating the C–terminal alanine of BhaA–Ala (BhaA^Ala40^) (Fig. 10b). Notably, the corresponding residues of Asp558, Glu813, and Arg815 in TglB (Glu542, Glu801, Arg803) have been experimentally shown to be critical for phosphorylation of the TglA C-terminus [59, 61, 63]. In stage 2, BhaB_7_ positions the phosphorylated Ala40 of BhaA–Ala and the –NH_2_ group of tryptophanyl moiety linked to tRNA^Trp^ near Arg34 (Fig. 10c), whose TglB counterpart (Arg10) has been verified as essential for cysteine installation onto TglA [59, 63]. In stage 3, the resulting BhaA–Ala–Trp–tRNA^Trp^ intermediate is hydrolyzed by water molecule likely exist in BhaB_7_ active site (Fig. 10d), releasing the extended BhaA–Ala–Trp peptide and free tRNA^Trp^ (Fig. 10e). Together, these modeled complexes provide preliminary structure–function insights into PEARL–catalyzed peptide C-terminal extensions (Fig. 10b-f).

## Conclusion

We first highlighted the discovery of the AlaRS-like enzyme AcmF in the biosynthesis of the aminoacyl nucleoside sulfamate ascamycin, achieved through a “Forecasting Biosynthesis” strategy that integrated bioinformatics, biochemical, and genetic approaches. This finding expanded the known repertoire of aa-tRNA-dependent enzymes, underscored the value of combining AI-based prediction with experimental validation in natural product research, and offered a promising chemoenzymatic platform for synthesizing diverse aminoacyl nucleoside sulfamates. Using AlphaFold 3-based complex modeling with aa-tRNAs and small molecule substrates, we further examined structure-function relationships across several enzyme families in this review. These analyses suggested several plausible catalytic strategies, including i) the active-site water-mediated proton-relay mechanisms used by the class II aaRS–like AcmF and class I aaRS–like SbzA in SB–203208 biosynthesis, ii) the substrate-assisted proton shuttling mechanism employed by members of GNAT–fold transferases in natural product biosynthesis, iii) the conserved ping-pong chemistry of CDPSs in cyclodipeptide assembly, and iv) the ATP- and aa-tRNA-dependent C-terminal peptide extensions catalyzed by LanB–like PEARLs. Together, these results deepen our understanding of aa-tRNA-dependent enzymology and highlight opportunities to harness these enzymes as versatile chemoenzymatic platforms for the synthesis of aminoacyl-containing molecules. Beyond the mechanistic advances presented here, this work has broader implications for enzyme engineering, natural product diversification, and the discovery of novel tRNA–dependent biocatalysts. While higher-resolution approaches such as X-ray crystallography and molecular dynamics simulations will be crucial for fully validating our proposed mechanisms, several additional methods could further strengthen structural and catalytic inferences. These include cryo–EM for visualizing large or flexible enzyme-tRNA assemblies; carbene-footprinting mass spectrometry to map substrate interactions and conformational changes; ^1^H–^15^N heteronuclear single quantum correlation NMR spectroscopy of tRNA upon protein binding to monitor structural perturbations; site-directed mutagenesis and pH-rate profiling to probe key catalytic residues and protonation states; and quantum mechanics/molecular mechanics calculations to assess transition states and reaction energetics. Integrating these orthogonal experimental and computational strategies will enable more comprehensive validation of AlphaFold-derived models and deepen our mechanistic understanding of aa-tRNA-dependent enzymatic transformations.

## References

[1] Lucas MC, Pryszcz LP, Medina R, Milenkovic I, Camacho N, Marchand V, et al. Quantitative analysis of tRNA abundance and modifications by nanopore RNA sequencing. Nat Biotechnol. 2024;42:72–86. 37024678 10.1038/s41587-023-01743-6 PMC10791586

[2] Kumar S, Das M, Hadad CM, Musier-Forsyth K. Substrate and enzyme functional groups contribute to translational quality control by bacterial prolyl-tRNA synthetase. J Phys Chem B. 2012;116:6991–9. 22458656 10.1021/jp300845h PMC3376218

[3] Moutiez M, Belin P, Gondry M. Aminoacyl-tRNA-utilizing enzymes in natural product biosynthesis. Chem Rev. 2017;117:5578–618. 28060488 10.1021/acs.chemrev.6b00523

[4] Maruyama C, Hamano Y. tRNA-dependent amide bond-forming enzymes in peptide natural product biosynthesis. Curr Opin Chem Biol. 2020;59:164–71. 32898755 10.1016/j.cbpa.2020.08.002

[5] Hu Z, Awakawa T, Ma Z, Abe I. Aminoacyl sulfonamide assembly in SB-203208 biosynthesis. Nat Commun. 2019;10:184. 30643149 10.1038/s41467-018-08093-x PMC6331615

[6] Zheng Y, Morita N, Takagi H, Shiozaki-Sato Y, Ishikawa J, Shin-ya K, et al. Alanyl-tRNA synthetase-like enzyme-catalyzed aminoacylation in nucleoside sulfamate ascamycin biosynthesis. ACS Catal. 2024;14:3533–42.

[7] Qin X, Hao Z, Tian Q, Zhang Z, Zhou C, Xie W. Cocrystal structures of glycyl-tRNA synthetase in complex with tRNA suggest multiple conformational states in glycylation. J Biol Chem. 2014;289:20359–69. 24898252 10.1074/jbc.M114.557249 PMC4106348

[8] Abramson J, Adler J, Dunger J, Evans R, Green T, Pritzel A, et al. Accurate structure prediction of biomolecular interactions with AlphaFold 3. Nature. 2024;630:493–500. 38718835 10.1038/s41586-024-07487-w PMC11168924

[9] Krishna R, Wang J, Ahern W, Sturmfels P, Venkatesh P, Kalvet I, et al. Generalized biomolecular modeling and design with RoseTTAFold All-Atom. Science. 2024;384:eadl2528.38452047 10.1126/science.adl2528

[10] Isono K, Uramoto M, Kusakabe H, Miyata N, Koyama T, Sethi SK, et al. Ascamycin and dealanylascamycin, nucleoside antibiotics from *Streptomyces* sp. J Antibiot. 1984;37:670–2. 10.7164/antibiotics.37.670 6547710

[11] Takahashi E, Beppu T. A new nucleosidic antibiotic AT-265. J Antibiot. 1982;35:939–47. 10.7164/antibiotics.35.939 7142012

[12] Osada H, Isono K. Mechanism of action and selective toxicity of ascamycin, a nucleoside antibiotic. Antimicrob Agents Chemother. 1985;27:230–3. 2580481 10.1128/aac.27.2.230 PMC176244

[13] Zhao C, Qi J, Tao W, He L, Xu W, Chan J, et al. Characterization of biosynthetic genes of ascamycin/dealanylascamycin featuring a 5’-*O*-sulfonamide moiety in *Streptomyces* sp. JCM9888. PLoS One. 2014;9:e114722. 25479601 10.1371/journal.pone.0114722 PMC4257720

[14] Chen Y, Lowe PT, Deng H, O’Hagan D. Halogenated adenine and adenosine natural products in *Streptomyces* sp. JCM9888. Eur J Org Chem. 2024;27:e202400578.

[15] Mirdita M, Schutze K, Moriwaki Y, Heo L, Ovchinnikov S, Steinegger M. ColabFold: making protein folding accessible to all. Nat Methods. 2022;19:679–82. 35637307 10.1038/s41592-022-01488-1 PMC9184281

[16] Naganuma M, Sekine S, Chong YE, Guo M, Yang XL, Gamper H, et al. The selective tRNA aminoacylation mechanism based on a single G*U pair. Nature. 2014;510:507–11. 24919148 10.1038/nature13440 PMC4323281

[17] Swairjo MA, Schimmel PR. Breaking sieve for steric exclusion of a noncognate amino acid from active site of a tRNA synthetase. Proc Natl Acad Sci USA. 2005;102:988–93. 15657145 10.1073/pnas.0409024102 PMC545860

[18] Guo M, Chong YE, Shapiro R, Beebe K, Yang XL, Schimmel P. Paradox of mistranslation of serine for alanine caused by AlaRS recognition dilemma. Nature. 2009;462:808–12. 20010690 10.1038/nature08612 PMC2799227

[19] Chong YE, Guo M, Yang XL, Kuhle B, Naganuma M, Sekine SI, et al. Distinct ways of G:U recognition by conserved tRNA binding motifs. Proc Natl Acad Sci USA. 2018;115:7527–32. 29967150 10.1073/pnas.1807109115 PMC6055181

[20] Polikanov YS, Steitz TA, Innis CA. A proton wire to couple aminoacyl-tRNA accommodation and peptide-bond formation on the ribosome. Nat Struct Mol Biol. 2014;21:787–93. 25132179 10.1038/nsmb.2871 PMC4156881

[21] Minajigi A, Francklyn CS. RNA-assisted catalysis in a protein enzyme: The 2’-hydroxyl of tRNA^Thr^ A76 promotes aminoacylation by threonyl-tRNA synthetase. Proc Natl Acad Sci USA. 2008;105:17748–53. 18997014 10.1073/pnas.0804247105 PMC2584683

[22] Takahashi A, Kurasawa S, Ikeda D, Okami Y, Takeuchi T. Altemicidin, a new acaricidal and antitumor substance. I. Taxonomy, fermentation, isolation and physico-chemical and biological properties. J Antibiot. 1989;42:1556–61. 10.7164/antibiotics.42.1556 2584137

[23] Takahashi A, Kurasawa S, Ikeda D, Okami Y, Takeuchi T. Altemicidin, a new acaricidal and antitumor substance. II. Structure determination. J Antibiot. 1989;42:1562–6. 10.7164/antibiotics.42.1562 2584138

[24] Stefanska AL, Cassels R, Ready SJ, Warr SR. SB-203207 and SB-203208, two novel isoleucyl tRNA synthetase inhibitors from a *Streptomyces* sp. I. Fermentation, isolation and properties. J Antibiot. 2000;53:357–63. 10.7164/antibiotics.53.357 10866217

[25] Houge-Frydrych CSV, Gilpin ML, Skett PW, Tyler JW. SB-203207 and SB-203208, two novel isoleucyl tRNA synthetase inhibitors from a *Streptomyces* sp. II. Structure determination. J Antibiot. 2000;53:364–72. 10.7164/antibiotics.53.364 10866218

[26] Yan Y, Liu N, Tang Y. Recent developments in self-resistance gene-directed natural product discovery. Nat Prod Rep. 2020;37:879–92. 31912842 10.1039/c9np00050j PMC7340575

[27] Silvian LF, Wang J, Steitz TA. Insights into editing from an Ile-tRNA synthetase structure with tRNA^Ile^ and mupirocin. Science. 1999;285:1074–7. 10446055

[28] Brkic A, Leibundgut M, Jablonska J, Zanki V, Car Z, Petrovic Perokovic V, et al. Antibiotic hyper-resistance in a class I aminoacyl-tRNA synthetase with altered active site signature motif. Nat Commun. 2023;14:5498. 37679387 10.1038/s41467-023-41244-3 PMC10485003

[29] Garg RP, Qian XL, Alemany LB, Moran S, Parry RJ. Investigations of valanimycin biosynthesis: elucidation of the role of seryl-tRNA. Proc Natl Acad Sci USA. 2008;105:6543–7. 18451033 10.1073/pnas.0708957105 PMC2373340

[30] Bougioukou DJ, Mukherjee S, van der Donk WA. Revisiting the biosynthesis of dehydrophos reveals a tRNA-dependent pathway. Proc Natl Acad Sci USA. 2013;110:10952–7. 23776232 10.1073/pnas.1303568110 PMC3704017

[31] Zhang W, Ntai I, Kelleher NL, Walsh CT. tRNA-dependent peptide bond formation by the transferase PacB in biosynthesis of the pacidamycin group of pentapeptidyl nucleoside antibiotics. Proc Natl Acad Sci USA. 2011;108:12249–53. 21746899 10.1073/pnas.1109539108 PMC3145694

[32] Maruyama C, Niikura H, Izumikawa M, Hashimoto J, Shin-Ya K, Komatsu M, et al. tRNA-dependent aminoacylation of an amino sugar intermediate in the biosynthesis of a streptothricin-related antibiotic. Appl Environ Microbiol. 2016;82:3640–8. 27084005 10.1128/AEM.00725-16 PMC4959175

[33] Yamato M, Iinuma H, Naganawa H, Yamagishi Y, Hamada M, Masuda T, et al. Isolation and properties of valanimycin, a new azoxy antibiotic. J Antibiot. 1986;39:184–91. 10.7164/antibiotics.39.184 3754251

[34] Johnson R, Kastner R, Larsen S, Ose E. A54556 Antibiotics and process for production thereof. In: USPTO ELaC, 14 (ed), 1984.

[35] Zhang W, Ostash B, Walsh CT. Identification of the biosynthetic gene cluster for the pacidamycin group of peptidyl nucleoside antibiotics. Proc Natl Acad Sci USA. 2010;107:16828–33. 20826445 10.1073/pnas.1011557107 PMC2947877

[36] Chen RH, Buko AM, Whittern DN, McAlpine JB. Pacidamycins, a novel series of antibiotics with anti-*Pseudomonas aeruginosa* activity. II. Isolation and structural elucidation. J Antibiot. 1989;42:512–20. 10.7164/antibiotics.42.512 2498264

[37] Ito Y, Ohashi Y, Sakurai Y, Sakurazawa M, Yoshida H, Awataguchi S, et al. New basic water-soluble antibiotics BD-12 and BY-81. II. Isolation Purif Prop J Antibiot. 1968;21:307–12. 10.7164/antibiotics.21.307 5701774

[38] Biarrotte-Sorin S, Maillard AP, Delettre J, Sougakoff W, Arthur M, Mayer C. Crystal structures of *Weissella viridescens* FemX and its complex with UDP-MurNAc-pentapeptide: insights into FemABX family substrates recognition. Structure. 2004;12:257–67. 14962386 10.1016/j.str.2004.01.006

[39] Benson TE, Prince D, Mutchler V, Curry K, Ho A, Sarver R, et al. X-ray crystal structure of *Staphylococcus aureus* FemA. Structure. 2002;10:1107–15. 12176388 10.1016/s0969-2126(02)00807-9

[40] Fonvielle M, Li de La Sierra-Gallay I, El-Sagheer AH, Lecerf M, Patin D, Mellal D, et al. The structure of FemX(Wv) in complex with a peptidyl-RNA conjugate: mechanism of aminoacyl transfer from Ala-tRNA(Ala) to peptidoglycan precursors. Angew Chem Int Ed Engl. 2013;52:7278–81. 23744707 10.1002/anie.201301411

[41] York A, Lloyd AJ, Del Genio CI, Shearer J, Hinxman KJ, Fritz K, et al. Structure-based modeling and dynamics of MurM, a *Streptococcus pneumoniae* penicillin resistance determinant present at the cytoplasmic membrane. Structure. 2021;29:731–42.e736. 33740396 10.1016/j.str.2021.03.001 PMC8280954

[42] Fields RN, Roy H. Deciphering the tRNA-dependent lipid aminoacylation systems in bacteria: Novel components and structural advances. RNA Biol. 2018;15:480–91. 28816600 10.1080/15476286.2017.1356980 PMC6103681

[43] Fonvielle M, Chemama M, Lecerf M, Villet R, Busca P, Bouhss A, et al. Decoding the logic of the tRNA regiospecificity of nonribosomal FemX(Wv) aminoacyl transferase. Angew Chem Int Ed Engl. 2010;49:5115–9. 20572225 10.1002/anie.201001473

[44] Ulrich EC, Bougioukou DJ, van der Donk WA. Investigation of amide bond formation during dehydrophos biosynthesis. ACS Chem Biol. 2018;13:537–41. 29303545 10.1021/acschembio.7b00949 PMC5856630

[45] Jacques IB, Moutiez M, Witwinowski J, Darbon E, Martel C, Seguin J, et al. Analysis of 51 cyclodipeptide synthases reveals the basis for substrate specificity. Nat Chem Biol. 2015;11:721–7. 26236937 10.1038/nchembio.1868

[46] Lautru S, Gondry M, Genet R, Pernodet JL. The albonoursin gene cluster of *S. noursei*: biosynthesis of diketopiperazine metabolites independent of nonribosomal peptide synthetases. Chem Biol. 2002;9:1355–64. 12498889 10.1016/s1074-5521(02)00285-5

[47] Gondry M, Sauguet L, Belin P, Thai R, Amouroux R, Tellier C, et al. Cyclodipeptide synthases are a family of tRNA-dependent peptide bond-forming enzymes. Nat Chem Biol. 2009;5:414–20. 19430487 10.1038/nchembio.175

[48] Harding CJ, Sutherland E, Hanna JG, Houston DR, Czekster CM. Bypassing the requirement for aminoacyl-tRNA by a cyclodipeptide synthase enzyme. RSC Chem Biol. 2021;2:230–40. 33937777 10.1039/d0cb00142b PMC8084100

[49] Yao T, Liu J, Liu Z, Li T, Li H, Che Q, et al. Genome mining of cyclodipeptide synthases unravels unusual tRNA-dependent diketopiperazine-terpene biosynthetic machinery. Nat Commun. 2018;9:4091. 30291234 10.1038/s41467-018-06411-x PMC6173783

[50] Bourgeois G, Seguin J, Babin M, Belin P, Moutiez M, Mechulam Y, et al. Structural basis for partition of the cyclodipeptide synthases into two subfamilies. J Struct Biol. 2018;203:17–26. 29505829 10.1016/j.jsb.2018.03.001

[51] Bourgeois G, Seguin J, Babin M, Gondry M, Mechulam Y, Schmitt E. Structural basis of the interaction between cyclodipeptide synthases and aminoacylated tRNA substrates. RNA. 2020;26:1589–602. 32680846 10.1261/rna.075184.120 PMC7566563

[52] Sutherland E, Harding CJ, Czekster CM. Active site remodelling of a cyclodipeptide synthase redefines substrate scope. Commun Chem. 2022;5:101. 36518199 10.1038/s42004-022-00715-2 PMC7613923

[53] Vetting MW, Hegde SS, Blanchard JS. The structure and mechanism of the *Mycobacterium tuberculosis* cyclodityrosine synthetase. Nat Chem Biol. 2010;6:797–9. 20852636 10.1038/nchembio.440 PMC2957485

[54] Moutiez M, Schmitt E, Seguin J, Thai R, Favry E, Belin P, et al. Unravelling the mechanism of non-ribosomal peptide synthesis by cyclodipeptide synthases. Nat Commun. 2014;5:5141. 25284085 10.1038/ncomms6141

[55] Sauguet L, Moutiez M, Li Y, Belin P, Seguin J, Le Du MH, et al. Cyclodipeptide synthases, a family of class-I aminoacyl-tRNA synthetase-like enzymes involved in non-ribosomal peptide synthesis. Nucleic Acids Res. 2011;39:4475–89. 21296757 10.1093/nar/gkr027 PMC3105412

[56] Ting CP, Funk MA, Halaby SL, Zhang Z, Gonen T, van der Donk WA. Use of a scaffold peptide in the biosynthesis of amino acid-derived natural products. Science. 2019;365:280–4. 31320540 10.1126/science.aau6232 PMC6686864

[57] Ortega MA, Hao Y, Walker MC, Donadio S, Sosio M, Nair SK, et al. Structure and tRNA specificity of MibB, a lantibiotic dehydratase from actinobacteria Involved in NAI-107 biosynthesis. Cell Chem Biol. 2016;23:370–80. 26877024 10.1016/j.chembiol.2015.11.017 PMC4798866

[58] Ortega MA, Hao Y, Zhang Q, Walker MC, van der Donk WA, Nair SK. Structure and mechanism of the tRNA-dependent lantibiotic dehydratase NisB. Nature. 2015;517:509–12. 25363770 10.1038/nature13888 PMC4430201

[59] Zhang Z, van der Donk WA. Nonribosomal peptide extension by a peptide amino-acyl tRNA ligase. J Am Chem Soc. 2019;141:19625–33. 31751505 10.1021/jacs.9b07111 PMC6927032

[60] Daniels PN, Lee H, Splain RA, Ting CP, Zhu L, Zhao X, et al. A biosynthetic pathway to aromatic amines that uses glycyl-tRNA as nitrogen donor. Nat Chem. 2022;14:71–7. 34725492 10.1038/s41557-021-00802-2 PMC8758506

[61] Ramos Figueroa J, Zhu L, van der Donk WA. Unexpected transformations during pyrroloiminoquinone biosynthesis. J Am Chem Soc. 2024;146:14235–45. 38719200 10.1021/jacs.4c03677 PMC11117183

[62] Nguyen DT, Ramos-Figueroa JS, Vinogradov AA, Goto Y, Gadgil MG, Splain RA, et al. Aminoacyl-tRNA specificity of a ligase catalyzing non-ribosomal peptide extension. J Am Chem Soc. 2025;147:37893–8. 41066767 10.1021/jacs.5c12610 PMC12550832

[63] Ramos-Figueroa J, Liang H, van der Donk WA. Substrate recognition by a peptide-aminoacyl-tRNA ligase. Proc Natl Acad Sci USA. 2025;122:e2423858122. 40106349 10.1073/pnas.2423858122 PMC11962472

